# Variations in the rancid-flavor compounds of human breastmilk under general frozen-storage conditions

**DOI:** 10.1186/s12887-018-1075-1

**Published:** 2018-03-02

**Authors:** Hsiao-Ying Hung, Yu-Yun Hsu, Pei-Fang Su, Ying-Ju Chang

**Affiliations:** 1 Department of Nursing & Institute of Allied Health Sciences, College of Medicine, National Cheng Kung University, No. 1, Daxue Rd., East Dist., Tainan, Taiwan; 2 Department of Statistics, National Cheng Kung University, Tainan, Taiwan; 3 Nursing Department, National Cheng Kung University Hospital, Tainan, Taiwan

**Keywords:** Human milk, Frozen breastmilk, Rancid-flavor, Frozen storage, Milk lipolysis

## Abstract

**Background:**

Human breastmilk provides the best nutrition for infants. When women or infants have difficulties in breastfeeding directly, breastmilk is usually pumped and frozen for later use. However, while frozen, breastmilk may develop a rancid flavor, which induces infant feeding stress and raises the mothers’ concerns about the quality of frozen breastmilk. Nevertheless, few studies have investigated the variations in the compounds that cause the rancid flavor of breastmilk during frozen storage.

**Methods:**

A repeated-measures design was adopted to quantify the variations in rancid-flavor compounds, namely acid value (AV), total free fatty acids (FFAs), and short-and intermediate-chain FFAs of breastmilk during frozen storage. Breastmilk was obtained from ten healthy mothers of full-term infants and each milk sample was divided into three aliquots: fresh, 7-day frozen and 30-day frozen samples. The fresh samples were immediately analyzed, while the others were frozen in a domestic fridge within a temperature range of −15 to −18 °C and analyzed 7 and 30 days later.

**Results:**

The rancid-flavor compounds of the breastmilk, namely AV, total FFAs and intermediate-chain FFAs, significantly increased with storage time, all of which reached the sensory threshold for detecting the rancid flavor of milk. In addition, the FFAs of the breastmilk samples frozen for 7 days far exceeded the detection threshold for unpleased rancid flavor, while the 30-day samples were higher than the intolerable level for most people.

**Conclusions:**

This study revealed that the human breastmilk develops a rancid flavor during frozen storage. Therefore, we recommend that when infants refuse thawed milk, mothers can try to provide freshly expressed milk whenever possible or provide breastmilk frozen for less than 7 days. Future studies could explore the methods for slowing breastmilk lipolysis to maintain its fresh flavor.

## Background

Fresh human breastmilk is rich in nutrients and immune antibodies, making it the ideal food for infants [1]. However, sometimes breastmilk must be pumped and stored for later use, such as when infants are prematurely born or sick in the hospital; when mothers must return to work or separated away their babies for other reasons; or even when mothers simply want to maintain or increase their milk supply.

The recommendations for storing the expressed breastmilk vary depending on the storage conditions. The general storage guidelines suggests that breastmilk can be kept at room temperature lower than 26°C for 4–6 hours, stored in a refrigerator at temperatures colder than 4°C for 3–8 days, and in a freezer at temperatures −17~ −20 °C for at least 3 months [2, 3]. Accordingly, when breastmilk is expected to be consumed later than 3 to 8 days, freezing is a common practice for mothers handling their milk at home.

Breastmilk freezing is believed to be safe since the major nutrients are preserved and the growth of harmful bacteria is retarded [4, 5]. However, a study reported that feeding premature infants (postmenstrual age nearly 37 weeks) with milk frozen for approximately one month induces more stress responses, e.g., vomiting, coughing, and gagging, when compared with fresh milk [6]. Despite the lack of evidence indicating that frozen milk harms infants’ health, such feeding stresses inhibit infants from obtaining adequate breastmilk nutrition and raise mothers’ concerns about the quality and safety of frozen breastmilk.

The premature infants’ feeding-stress responses observed in that study were reported to be related to the rancid flavor of the frozen milk [7, 8], which was caused by the products of milk lipolysis [2]. Milk lipolysis refers to the milk lipids being hydrolyzed by lipase into free fatty acids (FFAs) and glycerol. Large amounts of FFAs accumulation, especially volatile short and intermediate-chain fatty acids (C4:0–C12:0), cause a rancid flavor in milk [9]. Generally, milk lipids exist in the form of fat globules of 0.1 to 20 μm in diameter, which are enclosed by a fat globule membrane. This membrane separates the fat globules from the lipase in the milk serum, thereby avoiding the occurrence of lipolysis. However, the freezing process results in the crystallization of milk lipids and damages the fat globule membrane, allowing milk lipolysis to occur [7, 9]. Previous studies have indicated that only when milk is stored at temperatures below −70 °C or pasteurized before freezing will the milk lipase activity be inhibited, which delays the process of milk lipolysis [10, 11]. Be that as it may, the temperature range of typical domestic freezer is generally −18°C to −20°C and human breastmilk frozen storage at home is not usually pasteurized before freezing, and so the rancid-flavor development of breastmilk due to lipolysis is generally inevitable under the typical frozen-storage regime.

Although previous studies have reported the occurrence of milk lipolysis during frozen storage [10], variations in the rancid-flavor compounds of milk under the general frozen-storage conditions have rarely been studied. Moreover, investigating the variations of rancid-flavor compounds in human breastmilk under general frozen-storage conditions is important to not only better understand the possible impacts on the levels of flavor in breastmilk, but also to verify the appropriateness of the general recommendations for storing breastmilk.

Because human breastmilk is a dynamic and live fluid, numerous endogenous and exogenous factors affect the levels of milk lipolysis, e.g., gestational age, length of lactation, time of milking, nutrition states, hormonal treatment, and mastitis [12–14]. Therefore, this study adopted a repeated-measures design to determine the variations in rancid-flavor compounds of breastmilk under general frozen-storage conditions.

## Methods

A repeated-measures study was conducted between April and September 2009 in Southern Taiwan. The study was approved by the institutional review board at a university hospital and informed consent was obtained before the research.

### Human breastmilk collection and storage

**Fig. 1:**
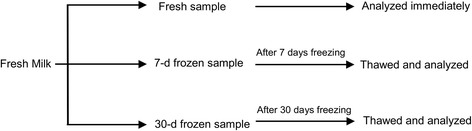
The process of handling each breast milk sample

Milk samples were obtained from ten healthy mothers of full-term infants with no mastitis, breast trauma, or abscesses, and no medication use one week prior to milk collection, which occurred between April 28 and May 26, 2009. The collection and storage procedures followed the general recommendations for handling human breastmilk for home use with health infants [2]. Milk was collected from one expression at the participating mothers’ homes. After being expressed, the milk samples were stored in sterile polyethylene milk bags and immediately refrigerated. Within 30 minutes of collection, the milk was transported to the laboratory in an insulated cooler filled with ice packs, which kept these samples at 4°C–6°C. Each participating woman provided nearly 150 ml in one pumping session. Because previous studies have indicated that longer periods of frozen storage causes more severe lipolysis in human breastmilk [15], each sample in this study was separated into three 50 ml aliquots and stored in glass bottles: one as the fresh sample (refrigerated for less than 24 hours), the second as the 7-day frozen sample, and the third as the 30-day frozen sample. The fresh samples were immediately analyzed. To emulate the general practice of women freezing their breastmilk at home, the 7-day and 30-day samples were frozen and stored in the back area in the freezer of a typical household fridge (freezer temperature range of −15°C to −18°C), which has separate doors for the refrigerator and freezer. After 7 and 30 days frozen storage, the frozen samples were completely thawed in the refrigerator for approximately 24 hours and then analyzed (Fig. 1).

### Human breastmilk analysis

The rancid-flavor compounds, acid value (AV), overall FFAs, and volatile short and intermediate-chain FFAs [9, 16] were analyzed in the university’s “Organic and Health food inspection laboratory”, which has certification from the Ministry of Health and Welfare of Taiwan.

Acid value is a hydrolytic rancidity indicator of milk fat. AV is defined as the number of milligrams of potassium hydroxide (KOH) required to neutralize the FFAs contained in one gram of fat [16]. To determine the AV of milk, the milk fat was firstly extracted via the Rose–Gottlieb method [17]. After the milk fat was extracted, a mixture of ethyl ether and ethanol (1:1, v/v) was used to dissolve it. Subsequently, two drop 1 % phenolphthalein was added as colorimetric indicator and then the mixture was titrated with a KOH ethanol solution. When pink color was observed for at least 10 seconds, the volume of KOH used was recorded. The AV was calculated according to the formula: Acid value =*V*_*KOH*_ × *M*_*KOH*_ × 56.1/*W*_*fat*_, *where V*_*KHO*_ is the volume of KOH used (ml), *M*_*KOH*_ is the molarity of the KOH solution, 56.1 is the molecular weight of KOH, *W*_*fat*_ is the milk fat in grams [16].

To analyze FFAs composition of milk, the processes of lipid extraction, lipid esterification, and gas chromatographic analysis were adapted. After milk fat was extracted via the Rose–Gottlieb method [17], two steps were then performed for the conversion of lipid-bound fatty acids and FFAs into fatty acid methyl esters (FAMEs) [18]. Heptane was used to dissolve the lipids after which sodium methoxide was added for transesterification of the glyceride-bound fatty acids into FAMEs. Hydrochloric acid then added to precipitate sodium glycerolate for the esterification of the FFAs into FAMEs. The resultant FAMEs were determined and quantified by gas chromatography (Shimadzu-17A, Tokyo, Japan) with a flame ionization detector and capillary column, which had an internal diameter of 0.53 mm × 30 mm and a l.0 mm film thickness.

### Statistical analysis

To address the limited number of studies and the different milk treatments used therein, the FFAs changes between the fresh and 7-day frozen breastmilk among the first five milk samples collected in the current study were used for power analysis. G-power [19] was applied to estimate the required sample size*.* The sample size was calculated based on the effect size, which was calculated through the formula:

$$ effect\ \mathrm{size}=\frac{mean\ {\mathrm{of}\ \mathrm{FFAs}}_{7\hbox{-} \mathrm{day}\ \mathrm{frozen}}- mean\ {\mathrm{of}\ \mathrm{FFAs}}_{\mathrm{fresh}}}{SD_{fresh}} $$

*Where* mean of FFAs 7 − day frozen = 12.68*,* mean of FFAs fresh=5.87*,* and SD fresh = 3.38). Given an effect size of 2.01and an alpha level of 0.05, a sample size of 10 for each group was necessary to achieve 80% power [20].

Data analyses included descriptive and inferential statistics. Descriptive statistics, including estimated mean, range, standard deviation for continuous variables, as well as percentages and frequencies for categorical variables, were tabulated and presented. With inferential statistics, considering the small sample size, a nonparametric Friedman test [21], which evaluates differences in medians among more than two groups, was adopted to compare the differences in the levels of AV, overall FFAs and short- and intermediate-chain FFAs among the fresh, 7-day and 30-day frozen samples. In addition, Wilcoxon signed rank test [22] was then used as post-hoc test to determine which pairs of milk samples differ. Moreover, the characteristics of FFA based on different groups (fresh, 7-day frozen and 30-day frozen) were graphically displayed using box plots to visualize and compare the change of FFA values. All statistical tests were 2-sided and conducted with R version 3.3.1. [23] and statistical package for social sciences (SPSS) 17.0.

## Results

The milk samples were donated from 10 healthy women with a mean age of 31 (range: 27–35 years old) and 124 days mean lactation. Most milk samples were expressed by pump, the fat content of which in the freshly expressed samples was 2.8 **±** 0.70 % (Table 1).

**Table 1:** Characteristics of milk donors and samples (*N* = 10)

Item	Range	Mean **±** SD/%
Age (years)	27–35	31 ± 3.6
Length of lactation (days)	34–180	124 ± 54
Milking by pump (n)	8	80
Fat content in milk (%)	2.02–4.34	2.8 **±** 0.70

**Fig. 2:**
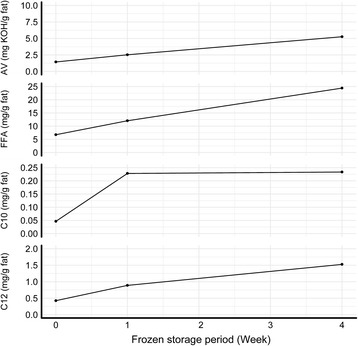
Variations of AV, overall FFAs, C10:0 and C12:0 during frozen storage. Mean is shown for each point. AV = acid value, FFAs = free fatty acids, C10 = caproic acid and C12 = lauric acid

In this study, the rancid-flavor compounds in breastmilk, AV, total FFAs, and two medium-chain FFAs, namely caproic acid (C10:0 ) and lauric acid (C12:0), were detected and all were found to increase with frozen-storage time. As shown in Fig. 2, the rate at which these compounds increased was seemingly faster between the fresh to 7 days frozen storage than that between the 7 to 30 days frozen storage.

**Fig. 3:**
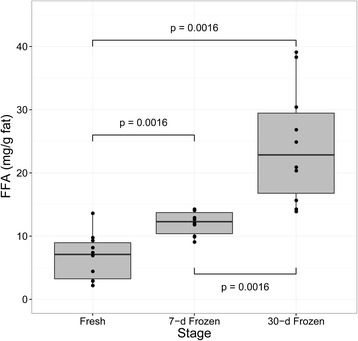
Distribution and comparison of overall FFAs levels among fresh and frozen samples. Black points represent FFAs level of each milk sample. For boxplot, the boundary of the box closest to zero indicates the 25th percentile, a line within the box marks the median and the boundary of the box farthest from zero indicates the 75th percentile. Friedman test is applied for comparing the differences among milk sample

As presented in Fig. 3, the Friedman test revealed significant differences in overall FFAs amounts among the fresh, 7-day, and 30-day frozen samples, with the post-hoc test showing that FFAs concentrations in the 30-day frozen samples were significantly higher than those in the fresh (Z = 2.803, *p* < 0.005) and 7-day frozen (Z = 2.803, *p <* 0.005) samples.

Similarly, Table 2 depicts that the concentrations of AV, caproic acid (C10:0), and lauric acid (C12:0) revealed significant differences between the fresh and frozen samples. The post-hoc analysis indicated that AV and the lauric acid (C12:0) levels in the 30-day frozen samples were significantly higher than in both the fresh samples (Z = 2.803, *p* =.005) and 7-day frozen samples (Z = 2.293, *p* =.022). The caproic acid concentration in the 30-day frozen sample was significantly higher than in the fresh sample (Z = 2.599, *p* =.009); however, no significant difference was found in the two other pairs (i.e. fresh vs. 7-day frozen, Z = 1.478, *p* = .139; 7-day vs. 30-day frozen, Z = 1.784, *p* = .074).

**Table 2.** Acid value, total FFAs, and medium-chain FFAs in fresh and frozen breastmilk (*N* = 10)

Composition	Fresh (1)	7-day Frozen (2)	30-day Frozen (3)	χ^2a^	*p* value	Post hoc^b^
	Mean ± SD	Mean ± SD	Mean ± SD			
AV (mg KOH/g fat)	1.43 ± 0.81	2.52 ± 0.44	5.26 ± 1.99	20.0	<.001	(2) > (1)**
						(3) > (1)**
						(3) > (2)**
FFAs (mg/g fat)	6.74 ± 3.64	12.03 ± 1.90	24.41 ± 9.25	20.0	< 0.001	(2) > (1)**
						(3) > (1)**
						(3) > (2)**
Medium-chain						
Caproic acid (C10:0)	0.05 ± 0.04	0.23 ± 0.49	0.23 **±** 0.15	10.4	0.006	(3) > (1)**
Lauric acid (C12:0)	0.43 ± 0.24	0.89 **±** 0.33	1.52 **±** 0.65	16.2	< 0.001	(2) > (1)**
						(3) > (1)**
						(3) > (2)*

*AV* Acid values, *FFAs* Free fatty acids

^a^Result of Friedman test; ^b^ Result of Wilcoxon signed rank test

*p < 0.05, **p < 0.01

## Discussion

This study revealed that the AV, total FFAs, and caproic acid (C10:0 ) and lauric acid (C12:0) of human breastmilk increased with storage time, and that concentrations in the 30-day frozen milk samples were significantly higher than those in the 7-day frozen and fresh milk samples. These findings indicate that breastmilk’s rancid-flavor progressively developed with frozen-storage time.

Nevertheless, few studies have considered the flavor of human breastmilk; in contrast, the flavor quality of cow’s milk is strongly emphasized in the dairy industry since it affects customer acceptance and, in turn, the earnings of dairy producers [24]. Therefore, to characterize the potential influence of the levels of FFAs, caproic acid, and lauric acid on the flavor of breastmilk, a comparison with the recommended sensory threshold of rancid flavor in dairy products was made. Early studies in dairy science have reported the relative levels of FFAs, caproic acid (C10:0), and lauric acid (C12:0) in terms of the sensory threshold for detecting the rancid flavor of milk [9, 25, 26].The detectable threshold usually means the minimum levels of a certain flavor compound perceived by at least 50% of the adult panelists [27]; accordingly, the FFAS levels in good-tasting milk should be lower than such thresholds [28].

Due to different analytical methods and panelist training, previous studies have reported considerable variations in the FFAs levels required for the detection threshold of the rancid flavor in milk, with a range from 1 to 3.62 mEq/100 fat [9]. In response, the International Dairy Federation (1987) concluded that the FFAs levels for detecting the rancid-flavor threshold is normally between 1.5 to 2.0 mEq/100 fat for most adult consumers, with levels exceeding approximately 1.5 mmol/L reaching the unacceptable threshold [29, 30]. Specifically, the levels of caproic acid and lauric acid in milk required for reaching the rancid-flavor threshold were reported to be 7 and 8 ppm, respectively [24].

To compare with the aforementioned flavor thresholds, the values of AV and FFAs in this study were converted into the same units. The AV of fresh, 7-day frozen, and 30-day frozen milk samples in this study were approximately 2.55, 4.49, and 9.19 mEq/100g fat, respectively (unit conversion formula:1 mg KOH/g fat = 1 × 100 ÷ 56.1 mEq/100g fat; *where* 56.1 = molecular weight of KOH). In addition, the FFAs levels of the fresh, 7-day frozen, and 30-day frozen milk samples in this study were approximately 0.73, 1.32, and 2.63 mmolL^−1^, respectively (unit conversion formula:1mg/g fat = 1 × 28/260 mmolL^−1^; *where* 28 = g fat contained in per liter human breastmilk of this study, 260 = average molecular weight of FFAs estimated by the FFAs profile obtained in this study). These levels indicate that the flavor of fresh milk may already reach the rancid-flavor detection threshold, and that the flavor of frozen milk may easily exceed the unacceptable rancid-flavor threshold. When focusing on the representative compounds of rancid flavor, we found that caproic acid concentrations in fresh and frozen breastmilk seemed to be below the detection levels (approximate values: fresh: 1.4 ppm, 7-day frozen: 6.44 and 30-day frozen: 6.44 ppm; unit conversion formula : 1 mg/g fat = 1 × 28 ppm; *where* 28 = g fat contained in per liter human breastmilk of this study). In contrast, the lauric acid levels in the fresh and frozen breastmilk were much higher than the detection threshold for rancid flavor (approximate values: fresh: 12.04, 7-day frozen: 24.92, and 30-day frozen: 42.56 ppm).

Based on this comparison, this study found that the rancid flavor of the fresh breastmilk samples had already reached the rancid-flavor detection threshold for adults (2.55 mEq/100 fat); meanwhile, the FFAs levels in the 7-day (4.49 mEq/100 fat) samples far exceeded it and the 30-day frozen milk samples (2.63 ± 1.0 mmol/L) reached intolerance level. In addition, lauric acid, which has unclean and soapy flavor attributes [25], may be the main contributor to the rancid flavor of human breastmilk.

Our study revealed that human breastmilk seems to be susceptible to lipolysis, even when fresh, which accords with findings of other studies. For example, Lavine & Clark (1987) showed that the FFAs levels in fresh breastmilk were approximately 2.94 mEq/100g fat (0.23 ± 0.087 mg/ml) [15]. Slutzah [11] found that the FFAs concentrations in fresh breastmilk analyzed within 2.4±1.2 hours of expression were approximately 4.48 mEq/100g fat (0.35 mg/ml). Another more recent study also indicated that human milk appears to be more susceptible to changes in flavor than bovine milk after frozen storage [8]. Therefore, the principle of FFA levels being lower than the detection threshold of the rancid flavor in milk may not be appropriate for direct application in assessing human breastmilk flavor. Moreover, one point worth noting was that the sensory threshold of detecting the rancid flavor in milk was based on adult panelists. However, previous studies have shown that infants’ flavor sensitivity may differ from adults because even the protein hydrolysate formulas (PHF), which are perceived as extremely unpleasant for adults, are generally acceptable for infants younger than three months or those previously exposed to PHF [31]. This implies that age and learning experience affect infants’ acceptance of food flavors [32]. Therefore, despite rancid-milk flavor being described as foul and objectionable by adults [27], it may be acceptable by infants, especially those younger than three months old.

Based on our findings, we assume that the FFAs levels found in fresh breastmilk not only facilitate fat digestion for infants but also provide learning experiences for infants so as to facilitate becoming accustomed to the rancid flavor in frozen breastmilk. However, despite the lack of evidence indicating that milk lipolysis alters nutritive components in human breastmilk [3], it was noted that extreme lipolysis in breastmilk may increase the probability of infants refusing it, as demonstrated in Hung’s study [6]. Therefore, we recommend that when infants refuse thawed milk, mothers could try to provide freshly expressed breastmilk or breastmilk frozen less than 7 days. Concurrently, the traditional principle of breastmilk use, “use the oldest milk in the refrigerator or freezer first” might be best implemented only when it has been frozen for less than 7 days to avoid infant feeding stress. Moreover, we recommend future studies explore methods for decreasing the rate of breastmilk lipolysis to prolong its acceptable flavor. Although pasteurizing has been proposed to reduce milk lipolysis by inactivating the lipase [9, 11], the appropriate procedure for women to pasteurize their milk safely requires further study.

As a preliminary study exploring the rancid-flavor compounds variations of human breastmilk under general frozen-storage conditions, several limitations are acknowledged. The first is that to include more women to participate this study was not easy because providing breastmilk for study may affect their infants’ feeding. Therefore, only 10 women with 30 milk samples were included in this study, which may affect the generalization of the findings. The second is that a direct sensory-perception test was not conducted in this study; accordingly, an assessment of infants’ acceptable thresholds of the rancid flavor in frozen breastmilk was not performed. Future studies could combine observations of infants feeding behaviors with quantitative analysis of flavor compounds to determine infants’ acceptable threshold for the rancid flavor of breastmilk. A third limitation is that there are many other reactions that can cause abnormal flavors in milk, e.g. lipid oxidation and proteolysis [27]; however, this study preliminarily focused on variations in the levels of breastmilk lipolysis during frozen storage. To extend this work, future studies could apply organoleptic testing through a trained panel and also analyze the objective compounds to explore the effects of lipid oxidation or proteolysis on breastmilk flavor under general storage conditions.

## Conclusions

Human breastmilk lipolysis increases in tandem with frozen-storage duration, which intensifies the rancid flavor in breastmilk. Lipolysis of 7-day frozen breastmilk far exceeded the threshold for detecting rancid flavor in dairy products, while the lipolysis in the 30-day frozen milk reached the intolerable level. To prevent infant feeding stress, we suggest that human breastmilk frozen and stored under general conditions be preferably consumed within 7 days. Moreover, the traditional recommendation of breastmilk use, namely “use the oldest milk in the refrigerator or freezer first”, might be best implemented only when the breastmilk has been frozen for less than 7 days. Accordingly, since breastmilk flavor modulates infants’ intake of breastmilk, future studies should explore methods for slowing breastmilk lipolysis to maintain its fresh flavor and extend its storability.

## Abbreviations

AV: Acid value
FFAs: Free fatty acids
KOH: Potassium hydroxide
FAMEs: Fatty acid methyl esters
PHF: Protein hydrolysate formulas
